# Cloning of *TaeRF1* gene from Caucasian clover and its functional analysis responding to low-temperature stress

**DOI:** 10.3389/fpls.2022.968965

**Published:** 2022-12-20

**Authors:** Xiaomeng Zhang, Jingwen Jiang, Zewang Ma, Yupeng Yang, Lingdong Meng, Fuchun Xie, Guowen Cui, Xiujie Yin

**Affiliations:** College of Animal Science and Technology, Northeast Agricultural University, Harbin, China

**Keywords:** Caucasian clover, *TaeRF1*, overexpression, low-temperature stress, antioxidant enzyme

## Abstract

Low temperature (LT) is an important threat to the normal growth of plants. In this study, based on the full-length transcriptome sequencing results, the cold resistance genes were cloned from Caucasian clover with strong cold resistance. We cloned the CDS of *TaeRF1*, which is 1311 bp in length and encodes 436 amino acids. The molecular weight of the protein is 48.97 kDa, which had no transmembrane structure, and its isoelectric point (pI) was 5.42. We predicted the structure of *TaeRF1* and found 29 phosphorylation sites. Subcellular localization showed that *TaeRF1* was localized and expressed in cell membrane and chloroplasts. The *TaeRF1* gene was induced by stress due to cold, salt, alkali and drought and its expression level was higher in roots and it was more sensitive to LT. Analysis of transgenic A. thaliana plants before and after LT treatment showed that the *TaeRF1* gene enhanced the removal of excess H_2_O_2_, and increased the activity of antioxidant enzymes, thus improving the plant’s ability to resist stress. Additionally, the OE lines showed increased cold tolerance by upregulating the transcription level of cold-responsive genes (*CBF1*, *CBF2*, *COR15B*, *COR47*, *ICE1*, and *RD29A*). This study demonstrates that *TaeRF1* is actively involved in the responses of plants to LT stress. We also provide a theoretical basis for breeding and a potential mechanism underlying the responses of Caucasian clover to abiotic stress.

## Introduction

The normal growth and development of plants are severely limited by various abiotic stresses in nature (Julia and Claudia, 2012), among which low temperature (LT) is an important threat to the normal growth of plants and adversely affects their survival and reproduction (Min et al., 2020). LT stress causes dehydration of plant cells and tissues, changes in membrane lipids, production of reactive oxygen species (ROS), and degradation of some necessary macromolecules, such as polysaccharides, lipids, photosynthetic pigments, enzymes and nucleic acids (Kazemi-Shahandashti and Maali-Amiri, 2018), LT stress can decrease the rate of photosynthetic rate and the performance of antioxidant defense systems and can also cause imbalances in osmotic regulation and active oxygen metabolism (Andreas Theocharis et al., 2012). Plants have evolved different mechanisms to handle LT stress during the historical evolution process (Zhang et al, 2016; Zhang et al., 2021). Among them, the ICE1-CBF-COR signaling pathway related to cold acclimation in A.thaliana is considered to be the main pathway that endows plants with LT resistance. C-repeat/dehydration response element binding factors (CBF/DREB) are critical transcription factors that actively regulate the expression of downstream cold-responsive (COR) genes during cold stress (Stockinger et al., 1997). Cold-induced CBF genes specificity identification combined with the existed in the promoter of conservative C-repeat/dehydration response motif (CRT/DRE, CCGAC), thereby rapidly inducing the expression of CBF and stimulating the expression of cold response (COR) gene (Jaglo et al., 1998; Thomashow, 2010). COR encodes hydrophilic peptides that stabilize the plasma membrane and enhance cold resistance in plants (Gilmour et al., 1998). ICE1 (CBF expression inducer 1) is a major regulator of the expression of C-repeat binding factors (CBFs) and CORs, and plays a role in inducing CBFs expression through specific binding CBF promoters (CANNTG) at LT (Chinnusamy et al., 2003; Wang et al., 2022). Numerous genes are involved in plant cold resistance, but the identification of gene function to date is limited. More genes that affect cold resistance are still needed to guide the molecular breeding of cold-resistant crops, and using conventional breeding strategies to improve cold resistance is a challenging task.

Eukaryotic releasing factor 1 (eRF1) is a kind of translation terminator protein that combines termination codons and ribosomes, and protein synthesis is terminated when the translating ribosome encounters one of UAA, UAG or UGA (Brown et al., 2015; Kurilla et al., 2020). In eukaryotes, termination of mRNA translation is the final step in protein biosynthesis, and this process is controlled by three factors: polypeptide chain releasing factor eRF1 and eukaryotic releasing factor 3 (eRF3), and ribosomal cycling factor ABCE1 (Urakov et al., 2017). In previous studies, A. thaliana eRF1 proteins encoding genes (EeRF1-1, eRF1-2 and eRF1-3) have been functionally validated in mutant S. cerevisiae strains (Chapman and Brown, 2004). eRF1 is involved in the translation termination of specific cysteine glutamolysins in the endosperm of rice and plays a role in the conversion of glutamolysin mRNA into nascent polypeptides (Elakhdar et al., 2019), a process that leads to decreased glutaminolysis protein levels in rice (Ushijima et al., 2011). The expression of eRF1 is tightly controlled because its concentration determines the termination efficiency and frequency of translation read-through, and the protein recognizes termination signals and promotes the hydrolysis of peptidyl-tRNA ester bonds (Polina et al., 2013, Denis et al., 2018; Lashkevich et al., 2020). Histological analysis revealed a reduced cell height, ectopic lignification of some bast sieve cells and bundle-forming layer regions, enhanced lignification of interbundle fibers, and altered cell division in bundle-forming tufts, most of which were disorganized with enlarged laminae, demonstrating that eRF1 affects cell elongation and radial division in Arabidopsis (Anne Petsch et al., 2005). The role of eRF1 in other plants has yet to be investigated.

Caucasian clover *(Trifolium ambiguum* M. Bieb.) is a perennial leguminous plant with a long crown, relatively low growth and multiple branches with deep roots (Zhang et al., 2019). It is also the only perennial leguminous clover species with underground root tillers and strong clonal growth *via* rhizomes (Taylar and Smith, 1997). This species originates in the cold climates of the Russian Caucasus Mountains, eastern Turkey and northern Iran (Barneby et al., 1965) and has strong cold resistance, flooding resistance, drought resistance and grazing tolerance (Brummer and Moore, 2000). Our research group previously used RNA-Seq and PacBio high-throughput sequencing technology to sequence the Caucasian clover transcriptome (Yin et al., 2020). In this study, we analyzed the expression pattern of the *TaeRF1* gene in Caucasian clover for the first time, and speculated that it unctions under some abiotic stresses. The *TaeRF1* gene in Caucasian clover was herein cloned and bioinformatically analyzed. The trans-*TaeRF1* gene was then inserted into *A. thaliana via* an Agrobacterium-mediated method, and physiological indicators under LT stress were measured to assess gene function. This study revealed the genes that confer the LT tolerance of Caucasian clover, thereby providing a theoretical basis and technical support for the further selection and breeding of excellent forage grasses and laying the foundation for the molecular breeding of Caucasian clover.

## Materials and methods

### Plant materials and treatments

Caucasian clover (*Trifolium ambiguum* Bieb.) was provided by the College of Animal Science and Technology at Northeastern Agricultural University, while Ben’s tobacco (*Nicotiana benthamiana*) and Colombian wild-type (WT) Arabidopsis were obtained from Wuhan Boyuan Biotechnology Co. Caucasian clover was cultured at 26°C under a 12 h photoperiod, and seedlings aged 28 days (d) were subjected to 4°C, 150 mmol/L NaCl, 150 mmol/L NaHCO_3_ and 15% PEG-6000. The roots, stems and leaves of each stressed Caucasian clover plant were harvested after 0 (CK), 3, 6, 12, 24 and 48 h of treatment. Three biological replicates were performed per treatment.

### Cloning and expression analysis of the *TaeRF1* gene

The total RNA was extracted from Caucasian clover leaves using an Ultrapure RNA Kit (ComWin Biotech Corporation, Beijing, China). The cDNA template for reverse transcription PCR was synthesized using HiScript II Reverse Transcriptase (Vazyme, Nanjing, China). The primers used for cloning were designed by Primer 5 for cloning *TaeRF1* based on the results of transcriptome sequencing (**Table S1**). PCR amplification was performed using 2×Phanta^®^ Max Master Mix (Vazyme Biotech Co.) from Caucasian clover cDNA as a template. The amplified PCR products were detected by 1% agarose gel electrophoresis, and the target gene fragment was recovered by a Vazyme FastPure Gel DNA Extraction Mini Kit. The obtained 1311 bp full-length fragment was cloned into a 5minTM TA/Blunt-Zero Cloning vector (Vazyme Biotech Co.) and then subjected to DNA sequencing. Real-time fluorescence quantification was performed with cDNA as the template (**Table S1**). The qRT-PCR analysis was performed using ChanQ Universal SYBR qPCR Master Mix Kit, and relative gene expression levels were calculated by the 2^−ΔΔ^Ct method. Using AtActin as the internal reference, specific primers (**Table S1**) were used for qPCR analysis of key genes responding to LT stress (*AtCBF1*, *AtCBF2*, *AtCOR15B*, *AtICE1*, *AtRD29A* and *AtCOR47*).

### Bioinformatic analysis of the *TaeRF1* sequence

BLAST was used to search for homologous sequences of *TaeRF1*. DNAMAN was used for multiple alignment of amino acid sequences. To investigate the evolutionary relationships between *TaeRF1* in Caucasian clover and other species, phylogenetic analysis was performed with MEGA 5 based on the maximum likelihood method. The physicochemical properties (including molecular weight, pI) and instability coefficient) of the *TaeRF1* amino acid sequence were analyzed using ProtParam software. The transmembrane structural domain of *TaeRF1* was predicted using the transmembrane prediction server TMHM, and its secondary structure was analyzed using Predict Protein online software. Protein tertiary structures were predicted using the SWISS-MODEL online site. Protein structural domain analysis of the *TaeRF1* gene of Caucasian clover was performed using the MEME online website. *TaeRF1* protein phosphorylation sites were predicted using the NetPhos 3.1 website. The Protscale online website was used to analyze the hydrophilicity of the *TaeRF1* protein, and *TaeRF1* signaling peptides were predicted using Signal P 4.1. Subcellular localization analysis of the *TaeRF1* amino acid sequence was performed using the Predict Protein online website.

### Construction of vectors

The plasmid of DH5α bacterial solution with correct sequencing was used as the template, and the overexpression and transient expression primers (**Table S1**) containing protective bases and enzyme cutting sites were used for PCR amplification, and the inserted fragment was obtained after gel recovery. pBWA(V)BS-ccdB plasmids were linearized by double digestion with Bsa I and Eco31 I. Overexpression expression vector were obtained by recombination of the inserted fragments and vectors. pCAMBIA1300-35S-sGFP plasmids were linearized by double digestion with BamHI and SacI. And instantaneous expression vectors were obtained by recombination of the respective inserted fragments and vector. The vectors were transferred into Escherichia coli DH5α for culture, and the single colony was selected for PCR detection and sequence alignment to determine the correct. The plasmid with correct sequencing results was transformed into Agrobacterium EHA105 by freeze-thaw method, and then screened on the double-resistant YEB medium containing kanamycin and rifampicin. After 60 h culture, the single colony was selected and verified by PCR to determine the correct.

### Subcellular localization analysis of *TaeRF1*

The tobacco seeds were seeded in a mixture of vermiculite and nutrient soil. After the seeds germinated, each seedling was transplanted to a separate pot and watered daily for 1 month. The agrobacterium solution containing the transient expression vector was taken out of the -80°C, and the YEB liquid medium containing Kan and Lif was shaken to turbidity. The bacterial solution was centrifuged, discarded the supernatant and cleaned twice with a working solution (0.5 mol/L MES, 100 mmol/L acetyleugenone, 1 mol/L MgCl). The concentration was measured and diluted to OD600 = 0.2, then injected into the back of Nicotiana benthamiana tobacco leaves (avoiding the main vein) with a 1 mL syringe. The infected leaf tissue was obtained under dark conditions for 48 h, and the cellular localization of *TaeRF1*: GFP fusion protein was determined by laser microscope.

### Generation of transgenic Arabidopsis plants overexpressing *TaeRF1*

The floral dip method was employed to transfer the *TaeRF1* gene into Colombia-0. After harvesting the seeds, all seeds were grown on MS medium containing glyphosate. The surviving plants were transplanted into the soil for further cultivation. Glyphosate was sprayed again for screening and PCR detection of transgenic plants, and T0 Arabidopsis plants were obtained. Three transgenic lines were selected from the successfully identified eleven transgenic lines for planting and seed harvesting. T1-transformed *TaeRF1* overexpressed Arabidopsis plants were obtained after seed germination screening and PCR detection.

### Phenotypic identification

WT and transgenic Arabidopsis seeds were soaked in 75% ethanol for 1 min, sterilized with 10% NaClO for 10 min, and rinsed with sterile water 6 times. They were then germinated on 1/2 MS media at 23°C and 4°C. Changes in germination were measured. The 1/2 solid MS medium is composed of MS powder (2.22 g/L), sucrose (15 g/L), and agar powder (4.25 g/L) and the pH is 5.8. The protrusion of the radicle was considered the standard for the germination of seeds. After germination, the plants were moved to the incubator, and the root lengths of the 14d seedlings were measured. The petri dishes were stored vertically. To investigate the cold tolerance of transgenic lines, the seedlings were transplanted into soil and vermiculite, and the 30d plants were subjected to LT stress at 4°C. After treatment, the expression analysis of the cold-responsive genes was analyzed in leaf samples. The experiments were repeated three times.

### Physiological measurements

WT and transgenic Arabidopsis thaliana were cultured for different times (0, 24 and 72 h) under normal conditions and LT conditions, and the physiological indexes of plant leaves were measured. For the enzyme activity assays, SOD, POD and CAT kit (Keming, Suzhou, China) were used to measure the activity of superoxide dismutase (SOD), catalase (CAT) and peroxidase (POD). The index measurement method and calculation method refer to the manual. Malondialdehyde (MDA) content was determined by the thiobarbituric acid method, and proline (Pro) content was determined by the acid ninhydrin method.

### Statistical analyses

Data statistics and chart making are through Excel and Origin. SPSS software and Duncan multiple comparisons were used to analyze the differences.

## Results

### Cloning and phylogenetic analysis of the *TaeRF1*

**Figure 1:**
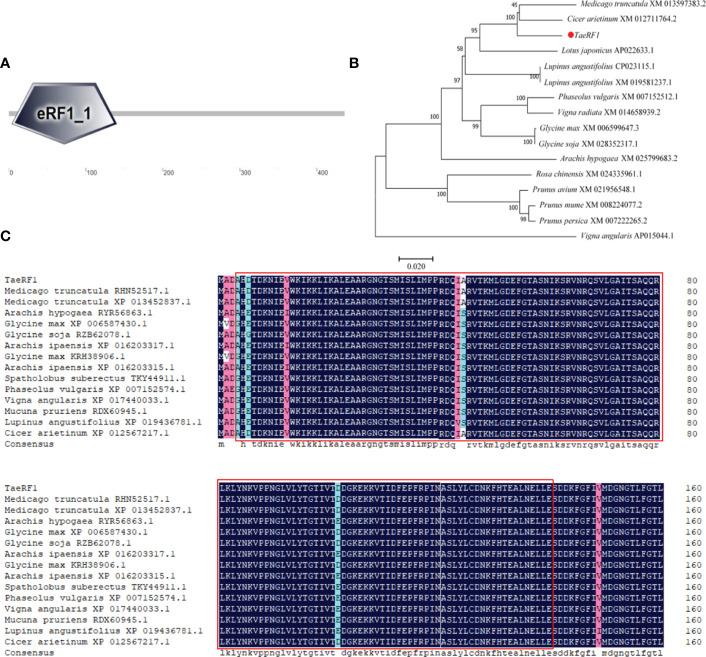
Cloning and multiple sequence alignment of *TaeRF1* homologous proteins. **(A)** SMART domain analysis of the *TaeRF1* gene. **(B)** Phylogenetic analysis of the *TaeRF1* gene in Caucasian clover. **(C)** Multiple sequence alignment of the eRF proteins. The color-coding indicates sequence similarity, with blue indicating the highest sequence similarity, pink indicating lower similarity, and cyan indicating the lowest similarity. The predicted conserved motifs of the eRF proteins are labeled as red square.

Total RNA of Caucasian clover was extracted, the quality of RNA was detected by Agarose gel electrophoresis (1% Agarose), and it showed bright and clear 18S and 28S bands (**Figure S1A**). The RNA was reversely transcribed into cDNA, and the quality was verified by PCR using reference primers (**Table S1**, **Figure S1B**). Specific primers were designed to clone *TaeRF1* CDS based on transcriptome data (**Table S1**). RT-PCR was used to amplify CDS of *TaeRF1* (**Figure S1C**), the amplified product was cloned into pCE2 TA/Blunt Zero vector and transferred to DH5α. Sequencing results showed that the CDS length of *TaeRF1* was 1311 bp, encoding 436 amino acids, and the nucleotide sequence and amino acid series were obtained (**Table S2**). The amino acid sequence of *TaeRF1* was uploaded to SMART website for domain prediction, and the results showed that *TaeRF1* contained an eRF1 domain between residues 4 and 140 (**Figure 1A**).

The *TaeRF1* protein sequence was subjected to a BLAST search using the NCBI protein website, and the results revealed high similarity to other eRF proteins. The amino acid sequence of the protein from Caucasian clover was aligned to those of its homologs in other organisms, and the phylogenetic tree was constructed by MEGA 5 (**Figure 1B**). (*Medicago truncatula* RHN52517.1, XP 013452837.1), peanut (*Arachis Hypogaea* RYR56863.1), soybean (*Glycine Max* XP 006587430.1), wild soybean (*Glycine Soja* RZB62078.1, KRH38906.1), Arachis ipaensis (XP 016203317.1, XP 016203315.1), pinto bean (*Spatholobus suberectus* TKY44911.1), kidney bean (*Phaseolus vulgaris* XP 007152574.1), cowpea (*Vigna angularis* XP 017440033.1), *Mucuna pruriens* (*Mucuna pruriens* RDX60945.1), lupin (*Lupinus angustifolius* XP 019436781.1), and chickpea (C*icer arietinum* XP 012567217.1), with similarities of 97.94%, 95.41%, 96.09%, 96.55%, 95.64%, 95.63%, 95.17%, 94.95%, 94.94%, 94.02% and 98.62%, respectively. The amino acid sequences were compared by DNAMAN and we label the eRF domain in the graph (**Figure 1C**). The *TaeRF1* gene was highly homologous to those from chickpea and *Medicago truncatula*, suggesting that the three genes are closely evolutionarily related and have similar functions.

### Physicochemical properties of the *TaeRF1* protein

The amino acid sequence of *TaeRF1* was analyzed by ProtParam. *TaeRF1* was shown to have a molecular formula of C_2172_ H_3411_ N_593_ O_672_ S_12_, a molecular weight of 48.97 kDa, and a theoretical pI of 5.42. The *TaeRF1* protein was mostly comprised of Leu residues (9.2%), and Trp and Cys residues accounted for the lowest percentage (both 0.7%). The protein contained 62 negatively charged residues and 53 positively charged residues. The protein was considered stable (stability coefficient 29.55), and the lipid index was 81.40.

To further analyze the *TaeRF1* protein, PredictProtein was used to predict its structure. The secondary structure of the *TaeRF1* protein was shown to comprise α-helices, β-extended strands and an irregular coil (**Figure S2A**). Among these features, irregular coil accounted for 46.56% of the protein, while α-helices and β-extended strands accounted for 35.55% and 17.89%, respectively. Swiss model predicted the tertiary structure of *TaeRF1* protein (**Figure S2B**).

The phosphorylation sites of the *TaeRF1* protein were predicted by NetPhos 3.1 website, revealing a total of 29 phosphorylation sites (**Figure S3A**), including 6 tyrosine phosphorylation sites, 7 threonine phosphorylation sites and 16 serine phosphorylation sites. The Protscale website was used to analyze the hydrophilicity and hydrophobicity of the *TaeRF1* protein (**Figure S3B**). The results revealed that most of the amino acids had negative values, and many negative amino acids have strong hydrophilic properties. Therefore, it can be inferred that *TaeRF1* is a hydrophilic protein. The trans-membrane domain of *TaeRF1* protein was predicted (S3C) by TMHMM online website, and the results showed that *TaeRF1* protein did not contain transmembrane domain (**Figure S3C**).

Signal P 4.1 was used to predict the signaling peptides of *TaeRF1*, yielding Max. Y and mean S values of 0.061 and 0.170, respectively. The fact that both of these values were less than 0.5 indicated that the *TaeRF1* protein had no signaling peptide cleavage sites and thus contained no signaling peptide sequences (**Figure S3D**).

### Subcellular localization analysis

**Figure 2:**
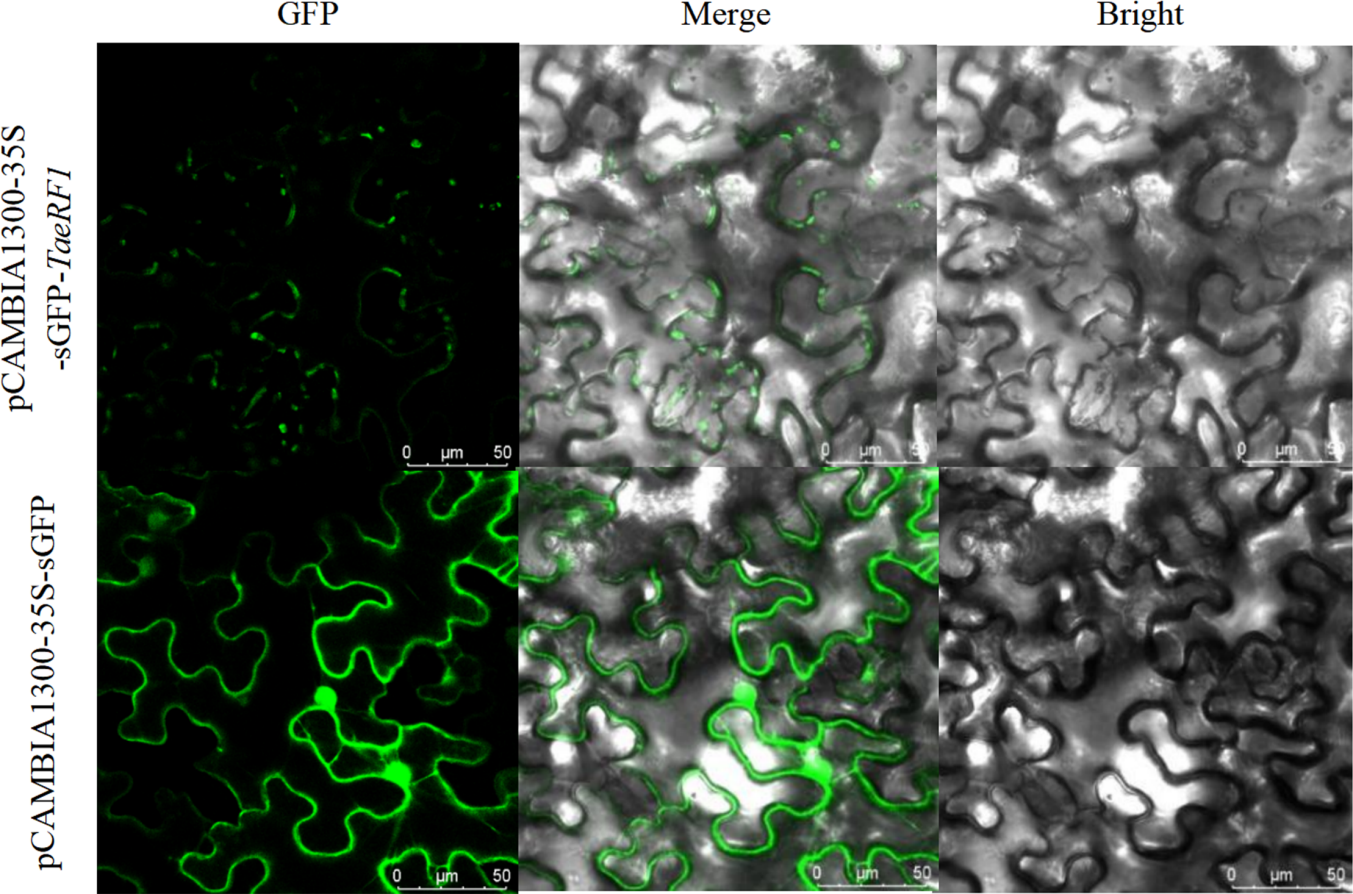
Subcellular localization of *TaeRF1*. Bright: bright-field; GFP: GFP Green fluorescent signal; Merged: merged images. Scale bar, 50 mm.

The localization of the protein encoded by the *TaeRF1* gene was therefore analyzed using the Predict Protein online website. The results suggested localization in the cytoplasm. To further assess the subcellular localization of *TaeRF1*, pCAMBIA1300-35S-sGFP plasmids were digested with BamHI and SacI, and the vectors were linearized. The target fragment was amplified with transient-expression primers, and the tobacco transient expression vector pCAMBIA1300-35S-sGFP-*TaeRF1* was obtained after recombination. The resulting tobacco transient expression vector construct was transformed into Agrobacterium EHA105, and the transformed cells were injected into tobacco. The transient expression of *TaeRF1* in *Nicotiana benthamiana* was observed by confocal laser microscopy, revealing expression in the cell membrane and chloroplasts (**Figure 2**).

### Analysis of the *TaeRF1* gene expression pattern

**Figure 3:**
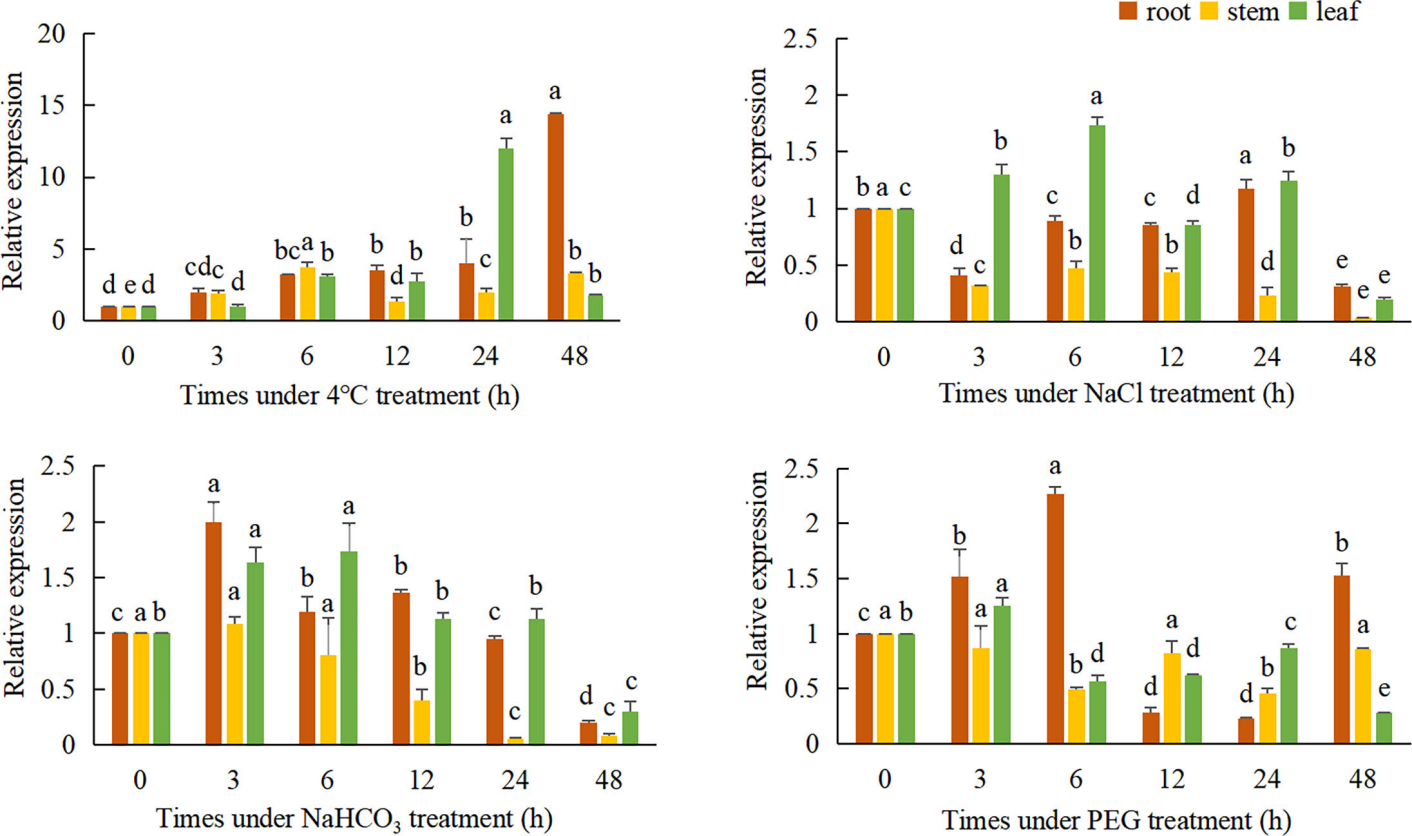
Expression of *TaeRF1* at various stress levels. Different characters on the error line represent a difference at a level of P<0.05 between the treatments. Different letters means significant difference at the 0.05 level.

QRT-PCR was used to detect the relative expression of the *TaeRF1* in roots, stems and leaves of Caucasian clover at different time points under LT, salt, alkaline and drought stress (**Figure 3**). The results revealed altered expression levels of *TaeRF1* changed in response to different stress treatments and at different time points. The variation in gene expression under the four stresses, salt (NaCl), alkalinity (NaHCO_3_), LT (4°C) and drought (15% PEG-6000), indicated that the gene responded to these four stresses.

Under LT stress, the expression level of *TaeRF1* gene was significantly increased, and the expression level was the lowest at 3 h and the highest at 48 h (*p<* 0.05). Stem expression of the *TaeRF1* gene was not obvious; the expression level was the highest at 6 h and the lowest at 12 h under stress, and the expression levels were significantly higher at these time points than at 0 h (*p<* 0.05). The expression level of the *TaeRF1* gene in leaves fluctuated, with the lowest expression observed at 3 h and the highest expression at 24 h (*p<* 0.05).

The expression level of *TaeRF1* gene in roots under 150 mmol/L NaCl stress increased firstly and then decreased, and reached the highest at 24 h (*p<* 0.05). The expression level of *TaeRF1* in the stems of the treated plants was significantly lower than that in the stems of CK plants (*p<* 0.05). The expression level of the *TaeRF1* gene in leaves was affected by salt stress: the highest value was observed at 6 h under stress and the lowest at 48 h, and the expression levels at 12 and 48 h were lower than that at 0 h (*p<* 0.05).

The expression of the *TaeRF1* gene in roots was affected under alkaline stress, initially exhibiting a decreasing trend, followed by an increase and then another decrease. The highest expression levels were observed at 3 h, while the lowest levels were observed at 48 h, and the levels were lower at 24 and 48 h than at 0 h. The expression levels at all other time points were higher than at 0 h (*p*< 0.05). The expression of *TaeRF1* in leaves was more obviously altered, initially showing an increasing trend, followed by a decrease. The highest expression level was observed at 6 h under stress, while the lowest levels were observed at 48 h under stress. The expression levels at 24 h and 48 h points were lower than that at 0 h. The expression levels at 24 h did not significantly differ from that at 0 h, while the levels at all other time points did significantly differ from that at 0 h (*p<* 0.05).

The expression of *TaeRF1* gene in the root under 15% PEG-6000 simulated drought stress was significantly higher than that in CK group at 6 h, and the lowest expression level was at 12 h and 24 h (*p*< 0.05). The expression of the *TaeRF1* gene in stems and leaves was not clear and showed a fluctuating trend.

### Generation and selection of *TaeRF1*-overexpressing transgenic A. thaliana plant lines

pBWA(V)BS-ccdB plasmids were linearized by double digestion with Bsa I and Eco31 I. The target gene was linked to the vector and introduced into agrobacterium tumefaciens receptive state. The transformed recombinant plasmid was identified by bacterial liquid PCR. A fragment of approximately 1300 bp was obtained, which was basically the same size as the *TaeRF1* gene (**Figure S4A**). Arabidopsis flowers were infected three times by Agrobacterium tumefaciens containing the target gene (**Figure S4B**). The mature seeds were collected and placed on petri dishes containing herbicides for culture. The surviving plants were transplanted into the soil for further culture, and herbicide was sprayed again for screening (**Figure S4C**). Eleven transgenic lines overexpressing *TaeRF1* were obtained, among which OE-1, OE-2 and OE-3 were significantly expressed (**Figure S4D**). Positive Arabidopsis plants were transplanted into soil for further culture until seeds were harvested (**Figure S4E**).

### Overexpression of *TaeRF1* in Arabidopsis improves tolerance to LT stress

**Figure 4:**
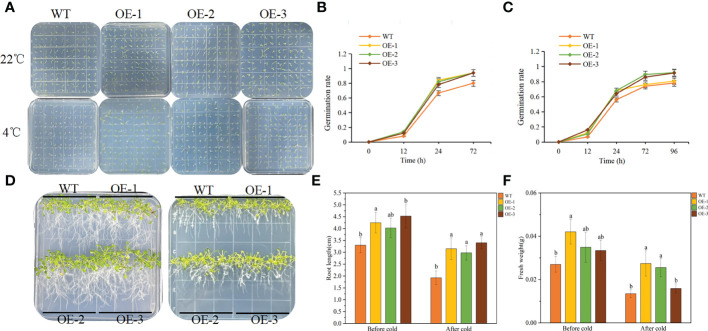
Overexpression of *TaeRF1* in Arabidopsis improved cold tolerance. Seed germination phenotype **(A)** and germination rate **(B-C)** of transgenic Arabidopsis lines overexpressing *TaeRF1* under LT stress. After 3 d of vernalization, one group was incubated at 22°C for 3 d (D left), and the other group was incubated at 4°C for 7 d (D right) and then moved to 22°C for 3 d. After that, the petri dishes were placed vertically. The root lengths and fresh weights of the 14-d-old seedlings were measured **(E–F)**. Different lowercase letters indicate that different plants showed a significant difference under the same stress (*p*< 0.05).

The germination experiment can explore the adaptation of *TaeRF1* to LT stress. WT and transgenic Arabidopsis seeds were planted on MS medium, and the germination rates was recorded at 23°C and 4°C. Under normal temperature (23°C), the germination rates of the transgenic seeds were similar to that of WT seeds. However, after moving to normal temperature (23°C) after LT (4°C) stress, seed germination of both the WT and the transgenic lines was inhibited (**Figure 4A**). The germination rates of transgenic seeds at 24 and 72 h were significantly higher than that of the WT (**Figures 4B, C**), and the root length and fresh weight after LT treatment were measured. At 23°C, the root length of transgenic seedlings was not much different from WT. Root length increased slowly at 4°C, but the difference was not significant, indicating that *TaeRF1* overexpression had a less obvious effect on root growth under low-temperature stress (**Figures 4D, E**). We measured the fresh weight and found that under LT treatment, the fresh weight of *TaeRF1*-overexpressing plants was significantly higher than that of WT, indicating that LT seriously affected the biomass of the plants (**Figure 4F**). Therefore, *TaeRF1* overexpression enhanced LT tolerance in transgenic seedlings.

### Functional verification of *TaeRF1*-overexpressing A. thaliana in response to LT stress

**Figure 5:**
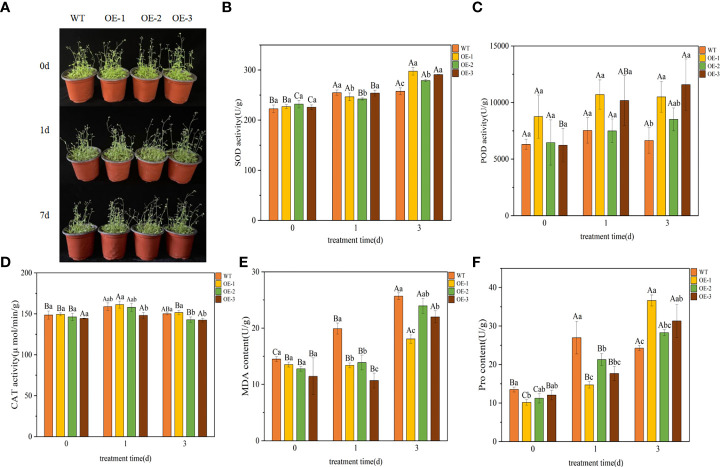
Functional verification of *TaeRF1*-overexpressing Arabidopsis under LT stress. Comparisons of phenotypes **(A)** and SOD, POD, CAT, MDA and Pro activities **(B-F)** between WT and transgenic *A. thaliana* under LT stress. The data are shown as the mean ± SD of three biological replicates. Different capital letters indicate that the same plant showed a significant difference on different stress days (*p<* 0.05); different lowercase letters indicate that different plants showed a significant difference under the same stress (*p<* 0.05).

We performed a LT stress (4°C) assay on 30 d plants of WT and transgenic plants to further characterize the phenotype of the transgenic lines. After 1 day of cold treatment at 4°C, the leaves of the plants turned dark green. After 3 d of being exposed to 4°C, transgenic plants showed mild wilting but grew well, while the WT plants showed obvious wilting and slow growth (**Figure 5A**). The effects of LT on plants can be further understood by measuring plant physiological indexes, the SOD, POD, CAT, MDA and Pro contents in plants were measured. There was no significant difference between transgenic and WT lines under normal conditions, but almost all plants were affected to some extent under LT stress condition.

Under LT stress, the SOD content in WT and transgenic plants overexpressing *TaeRF1* tended to be upregulated (**Figure 5B**). After 3 d of stress, the SOD content in transgenic plants was higher than that in the WT plants, and the difference was significant (*p<* 0.05). After 3 d of stress, the POD content in transgenic plants overexpressing *TaeRF1* was higher than that in WT plants (**Figure 5C**). The CAT content in transgenic and WT plants first increased and then decreased (**Figure 5D**), but changes in the CAT content in transgenic plants were not significant (*p<* 0.05). Under LT stress, the MDA content of WT increased compared with CK group (P< 0.05). The content of MDA in OE-2 increased continuously, the content of MDA in CK group was the lowest, and the content of MDA in OE-2 group was the highest after 3 d of stress treatment (P< 0.05). MDA content of OE-1 and OE-3 decreased firstly and then increased (*p<* 0.05). Under the same stress time, the content of MDA in CK group was the highest (**Figure 5E**). Under LT stress, Pro content of Arabidopsis and WT showed a trend of first increasing and then decreasing (**Figure 5F**). After LT stress for 3 d, the Pro content of transgenic plants was significantly higher (*p<* 0.05).

### *TaeRF1* activates the expression of LT stress-responsive genes

**Figure 6:**
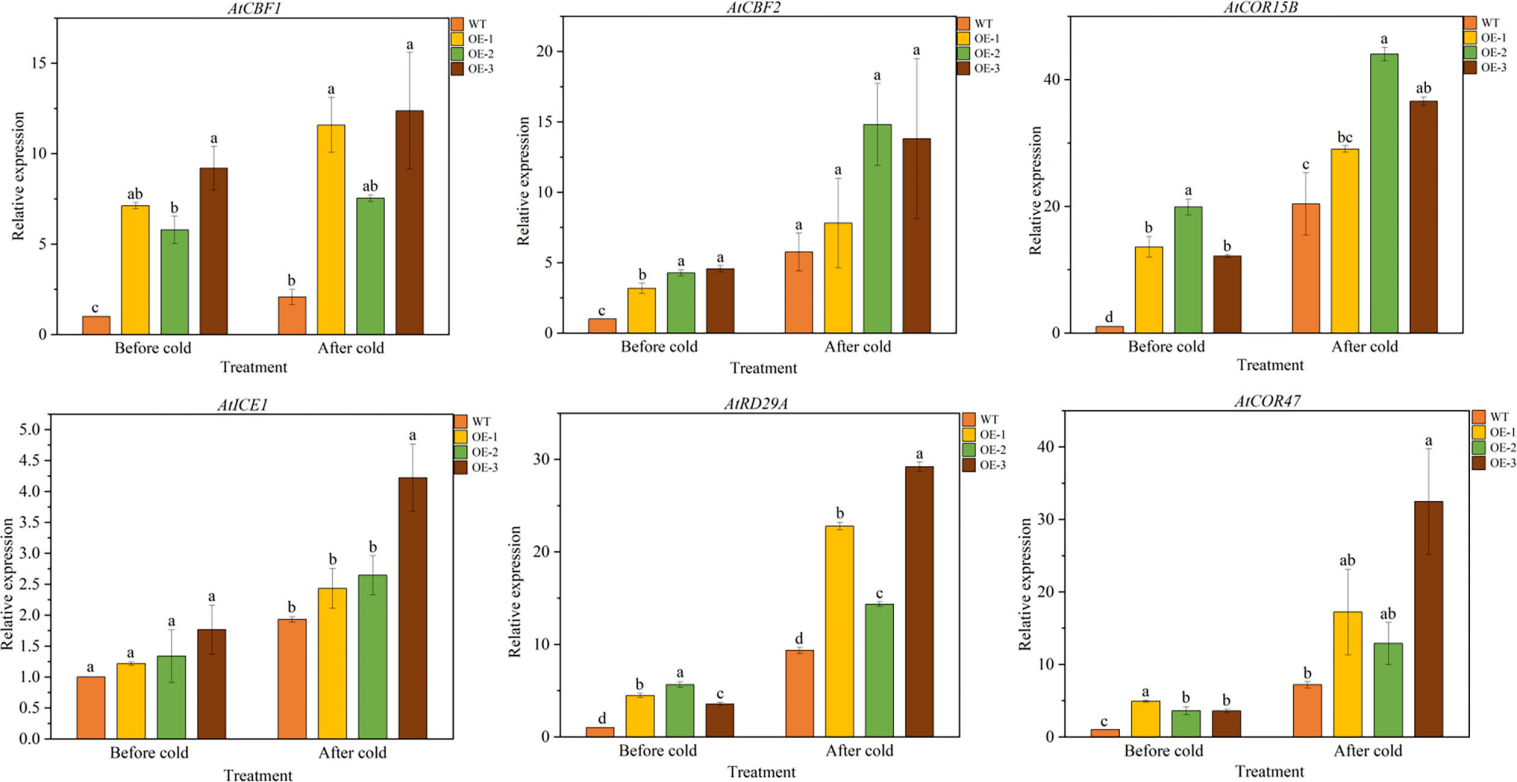
Expression patterns of cold-responsive genes in WT and *TaeRF1*-overexpressing Arabidopsis lines under cold stress treatment. Expression profiles of cold-responsive genes, including *AtCBF1*, *AtCBF2*, *AtCOR15B*, *AtICE1*, *AtRD29A* and *AtCOR47*. Different letters means significant difference at the 0.05 level.

We further analyzed the expression of LT responsive genes in Arabidopsis by qRT-PCR, such as *AtCBF1* and *AtCBF2* and their associated genes, including *AtCOR15B*, *AtICE1*, *AtCOR47* and *AtRD29A* (**Table S1**). The results showed that under normal conditions, there was no significant difference in the expression levels of 6 LT response genes between WT and overexpressed plants. After LT treatment, the expression level of six genes in overexpressed lines was significantly higher than that of WT, indicating that *TaeRF1* positively regulates the expression of corresponding genes in LT and can improve the cold tolerance of transgenic Arabidopsis (**Figures 6A–F**).

## Discussion

As a naturally stress-resistant species, the Caucasian clover represents an important resource for studying the resistance mechanisms and forage breeding. In this study, eRF1 gene was successfully cloned from the third-generation full-length transcriptome data of Caucasian clover. After cloning CDS, the sequencing results were consistent with the predicted results, and the gene was named TaeRF1. The full-length CDS of *TaeRF1* was determined to be 1311 bp, encoding 436 amino acids. SMART domain analysis showed that the *TaeRF1* gene has an eRF1 domain, which showed that the *TaeRF1*gene belongs to the eRF1 family. Phylogenetic tree analysis and multiple sequence alignment between the gene in Caucasian clover and homologs in other organisms showed that *TaeRF1* is highly homologous to eRF1 in *M. truncatula* and chickpea, which are also in the legume family. Analysis of the *TaeRF1* gene expression pattern showed that *TaeRF1* participates in the responses to several stresses such as cold, salinity, alkalinity, and drought. The roots of plants subjected to LT stress exhibited significantly increased *TaeRF1* gene expression, indicating that this gene may be involved in the response of plants to LT stress and that *TaeRF1* may perform functions in the root.

LT can prohibit plant growth, development, survival and productivity and is the most severe stress that limits normal plant growth (Mishra et al., 2018). In response to complex environmental changes, plants have evolved different signaling mechanisms to cope with stress. LT causes the accumulation of ROS, such as singlet oxygen, hydrogen peroxide and superoxide radicals, which can lead to cellular oxidative stress, a major factor underlying the damage caused by LT (Gómez et al., 2019). To scavenge ROS and reduce oxidative damage, plants have developed an effective defense system consisting of several antioxidant enzymes, such as catalase, ascorbate peroxidase, superoxide dismutase and peroxidase (Bai, 2019). Usually, upon the exposure of plants to abiotic stress, tolerant cells activate enzymatic antioxidant systems to remove ROS and protect the cells (Yu, 2020). At 48 h of LT stress, the relative expression of the *TaeRF1* gene significantly peaked in the roots and was 14.38-fold higher than that in the control plants, and its expression tended to increase. At 24 h of stress, the relative expression was more pronounced in the leaves, indicating that this gene can respond to LT stress. Caucasian clover grows in the extremely cold Caucasus Mountain region and therefore has a certain degree of cold tolerance and can overwinter. The high *TaeRF1* gene expression in the roots indicates that the roots play a positive role in resisting damage caused by LT. Moreover, ROS may be produced in leaf cells over periods of prolonged stress, resulting in increases in the levels of various peroxidases to eliminate ROS, thus increasing the cold tolerance of Caucasian clover.

Under adverse stress conditions, most plants accumulate large amounts of ROS, which damage the plant membrane system (Hu et al., 2010; Feng et al., 2014; Peng et al., 2019). The balance of intracellular ROS production and clearance can protect plants from stress damage. In plant cells, the SOD enzyme can eliminate ROS, functioning to eliminate and convert ${\text{O}}_{2}^{-}$ into H_2_O_2_ to limit plant cell damage (Wang, S. Q. et al., 2019; Lin et al., 2019). When WT plants were stressed for 1 day, SOD was used to eliminate ${\text{O}}_{2}^{-}$ and thereby prevent the excess accumulation of ROS. However, the SOD content was reduced after 3 d of stress in the WT plants, and the SOD activity decreased, while the SOD content in the transgenic plants overexpressing *TaeRF1* continued to increase, indicating that ROS was eliminated continuously under stress conditions and that plant cell damage was mitigated (Wang, Y. et al., 2019). At 3 d of LT stress, the SOD content was the highest in the OE-1 plants, and the SOD contents in the OE-1, OE-2 and OE-3 plants were significantly different from that in the WT plants (*p<* 0.05). In conclusion, the overexpression of *TaeRF1* may affect the SOD content under stress, thereby reducing plant damage; the *TaeRF1* gene can increase the SOD content under conditions of LT stress. These results also support the hypothesis that *TaeRF1* enhances stress tolerance by promoting the production of plant protective enzymes (Li et al., 2008).

Because excessive H_2_O_2_ is harmful to plant cells, POD is also indispensable for the protective enzyme system in plants, as it can degrade the generated H_2_O_2_ to limit damage to the membrane system (Sun et al., 2019, Surisa Phornvillay et al., 2019; Wang, M. et al., 2019). The result demonstrates that LT stress increased the POD content in the transgenic plants overexpressing *TaeRF1* to a certain extent. Compared with the WT plants, the transgenic plants had some certain resistance to LT stress. The *TaeRF1* gene may have a certain impact on the POD content under LT stress (Hao et al., 2018). Nearly all aerobic cells contain CAT, which functions similarly to POD, as it can degrade excessive H_2_O_2_ in plant tissues into oxygen and water, thereby reducing ROS accumulation in plant tissues (Han et al., 2020; Sun et al., 2020) and maintaining the integrity of the plant cell structure. To protect the plant cell membrane system, the browning of plant tissues and the aging of cells are delayed (Zhang et al., 2017). Under LT stress, the CAT contents in the WT and transgenic plants first increased and then decreased. After 1 day of stress, the contents in the OE-1 and OE-2 plants were comparable to that in the OE-3 plants, while the contents in the WT, OE-1, OE-2 and OE-3 plants were significantly different after 3 d of stress (*P*<0.05). Therefore, the enhanced antioxidant capacity of overexpressing *TaeRF1* in Arabidopsis can alleviate the damage of plants under LT stress.

LT can damage plants in many ways. The MDA content can reflect the degree of damage to the cell membrane, which is the first structure that is destroyed upon exposure to LT. MDA is a product of lipid peroxidation caused by ROS, which can inhibit the activity of protective enzymes and thus aggravate membrane lipid peroxidation (Liu et al., 2014; Sha et al., 2011; Wu et al., 2012). Studies have shown that the MDA content reflects the degree of damage to plant tissues under stress (Ning et al., 2021). The lower MDA content in transgenic plants under LT stress indicated less membrane damage than WT. The overexpression of TaeRF1 has a protective membrane integrity under LT stress. The amino acid Pro exists in a free state in plants. Under stress conditions such as drought, LT and salinity, Pro accumulates in large quantities in most plants. In addition to being an osmotic regulatory substance in the plant cytoplasm, Pro can stabilize the structures of biological macromolecules, reduce the acidity of plant cells and remove toxic ammonia (Polturak et al., 2018; Yan et al., 2019). In addition, Pro can regulate the ROS balance in plants and ensure balance in the cytoplasm. The membrane integrity and cold tolerance of plants are important, and Pro is a key factor underlying cold tolerance because it regulates osmotic pressure (Savoi et al., 2016; Zhao et al., 2019). Therefore, changes in Pro metabolism play an important role in improving the LT adaptability of plants. The Pro content in plants reflects their stress tolerance to a certain extent, and stronger LT tolerance is correlated with higher Pro accumulation (Wang, 2019). Under LT stress, the Pro content in WT plants was increased more than that of *TaeRF1*-overexpressing plants after 1 day of stress and decreased after 3 d of stress, while the Pro content in the transgenic plants continued to increase under stress, and the Pro contents of OE-1, OE-2 and OE-3 plants were significantly different from those in the WT plants after 3 d of stress (P< 0.05). These results indicate that the *TaeRF1* gene may resist the adverse effects of LT stress by increasing the Pro content.

The most classical plant response to cold stress is the ICE-CBF-COR regulatory pathway (Chinnusamy et al., 2007; Hwarari et al., 2022). This study quantitatively analyzed the expression levels of the CBF-dependent pathway, and their downstream cold stress responsive genes *AtCOR15B* and *AtCOR47*. We found that *TaeRF1* upregulates overexpressed Arabidopsis cold response genes. These experimental results were consistent with current expectations and similar to those of other studies (Dong et al., 2021; Yin et al., 2021). Therefore, we predicted that TaeRF1 likely enhances cold tolerance *via* the CBF-dependent pathway in Arabidopsis.

In this study, we cloned the *TaeRF1* gene from Caucasian clover, performed bioinformatics analyses to understand the genetic information, and analyzed the evolutionary relationships among multiple species. *TaeRF1* was transferred into Colombian Arabidopsis, and transgenic Arabidopsis plants overexpressing *TaeRF1* were successfully obtained. Physiological indicators were measured to assess the possible functions of the *TaeRF1* gene, and we speculated that *TaeRF1* was highly responsive to cold stress. The overexpression of *TaeRF1* in response to abiotic stress could regulate antioxidant enzyme activity in plants. In subsequent experiments, the gene can be transformed into Caucasian clover to obtain overexpression plants, and measurements can be performed to better understand its functions.

## Conclusions

In this study, the *TaeRF1* gene was screened and cloned based on the full-length transcriptome sequencing results of third-generation Caucasian clover plants. The expression of *TaeRF1* was significantly induced in response to LT stress. Overexpression of *TaeRF1* significantly enhanced LT resistance, and could better reduce the accumulation of ROS under cold stress in Arabidopsis thaliana. *TaeRF1* mediates cold signal transduction by increasing the transcription level of stress-responsive genes, thereby improving transgenic plant tolerance to cold stress. These results indicate that *TaeRF1* plays an active regulatory role in the responses of plants to LT stress. At the same time, *TaMYC2* in Caucasian clover can also increase the activity of antioxidant enzymes in plants, and increase the expression of ROS scavenging related genes and stress response genes under LT and drought stress, thereby enhancing the response ability of transgenic plants to stress (Zhao et al., 2022). Taken together, these findings provide further insights into the properties of *TaeRF1* protein and how it protects plants at low temperatures, and provide references for clover breeding.

## Data availability statement

The datasets presented in this study can be found in online repositories. The nucleotide sequence TaeRF1 can be found at GenBank with the accession number OQ000837. Further inquiries should be directed to the corresponding author.

## Author contributions

GC and XY conceived and designed the experiments; XZ and JJ performed the experiments; YY, ZM, LM, and FX analyzed the data; and XZ and JJ wrote the manuscript. All authors contributed to the article and approved the submitted version.
