# Supplementary material for: Cloning of TaeRF1 gene from Caucasian clover and its functional analysis responding to low-temperature stress
Source: Front Plant Sci. 2022 Dec 20;13:968965. doi: 10.3389/fpls.2022.968965 (PMC9809470; doi:10.3389/fpls.2022.968965)

**Figure S1.** RNA electrophoresis (A) cDNA quality assessment (B) of Caucasian clover and Results of PCR amplification of TaeRF1 gene (C).

18S

28S

750bp

2000bp

M 1 2 3 4 5 6 7


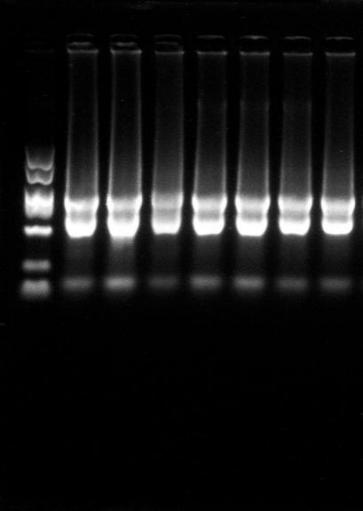


750bp


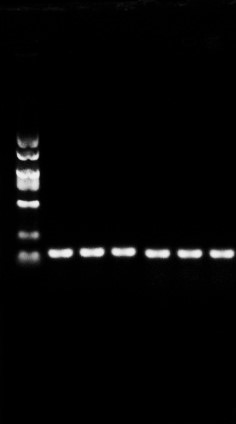


2000bp

M 1 2 3 4 5 6

2000bp

750bp

1311bp

M 1


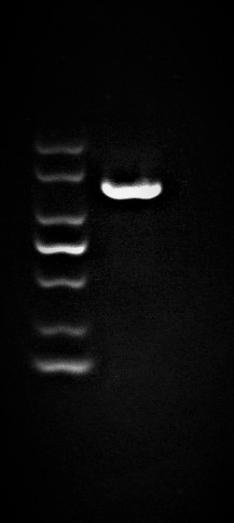


**C**

**A**

**B**


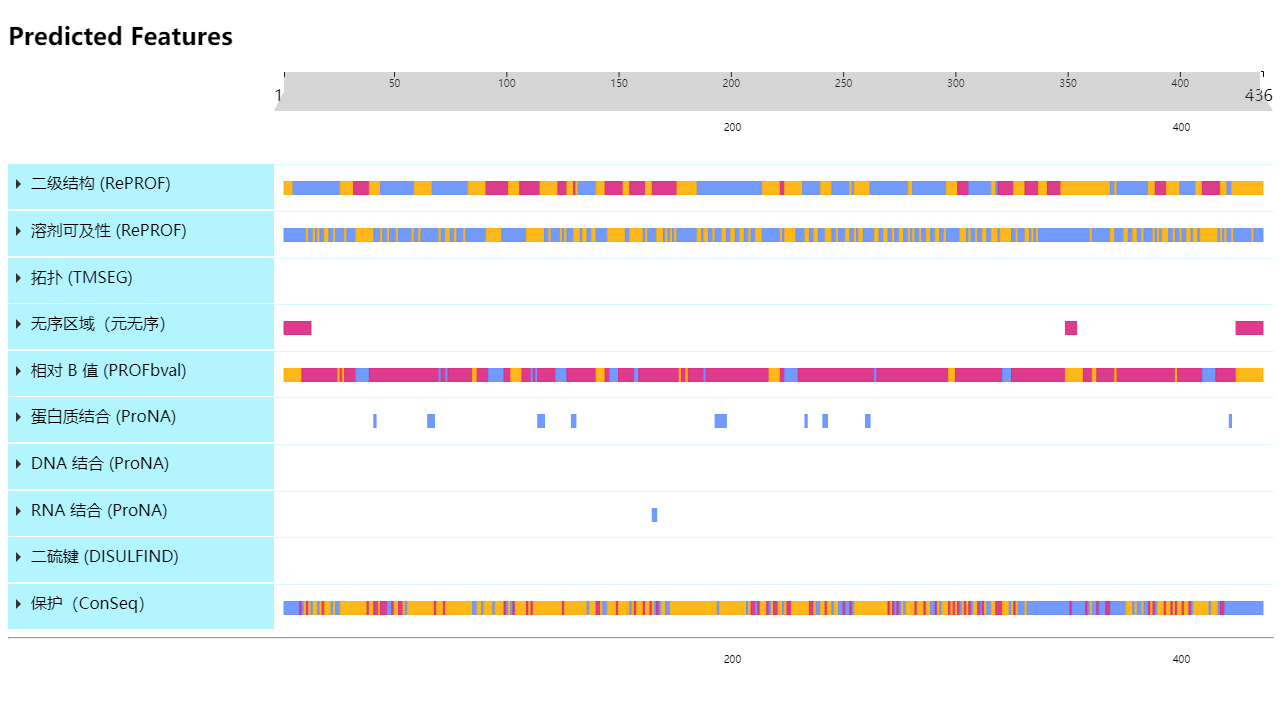


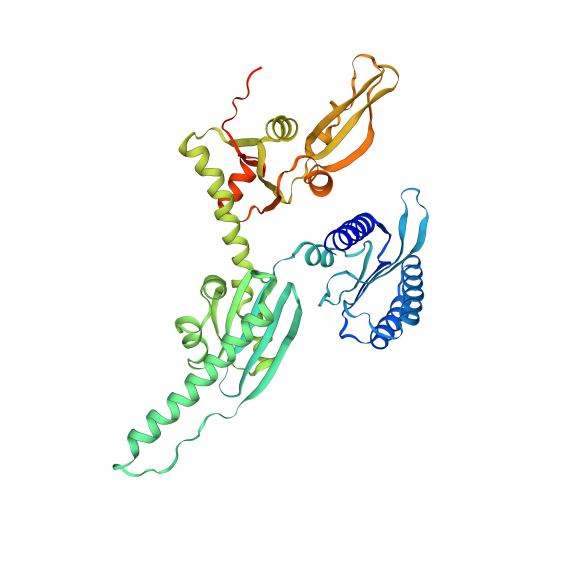
**Figure S2.**  Prediction of the TaeRF1 protein structure. (a) Prediction of the TaeRF1 protein secondary structure based on its complete amino acid sequence. (b) Prediction of the TaeRF1 protein tertiary structure based on its complete amino acid sequence.

**B**

**A**

**
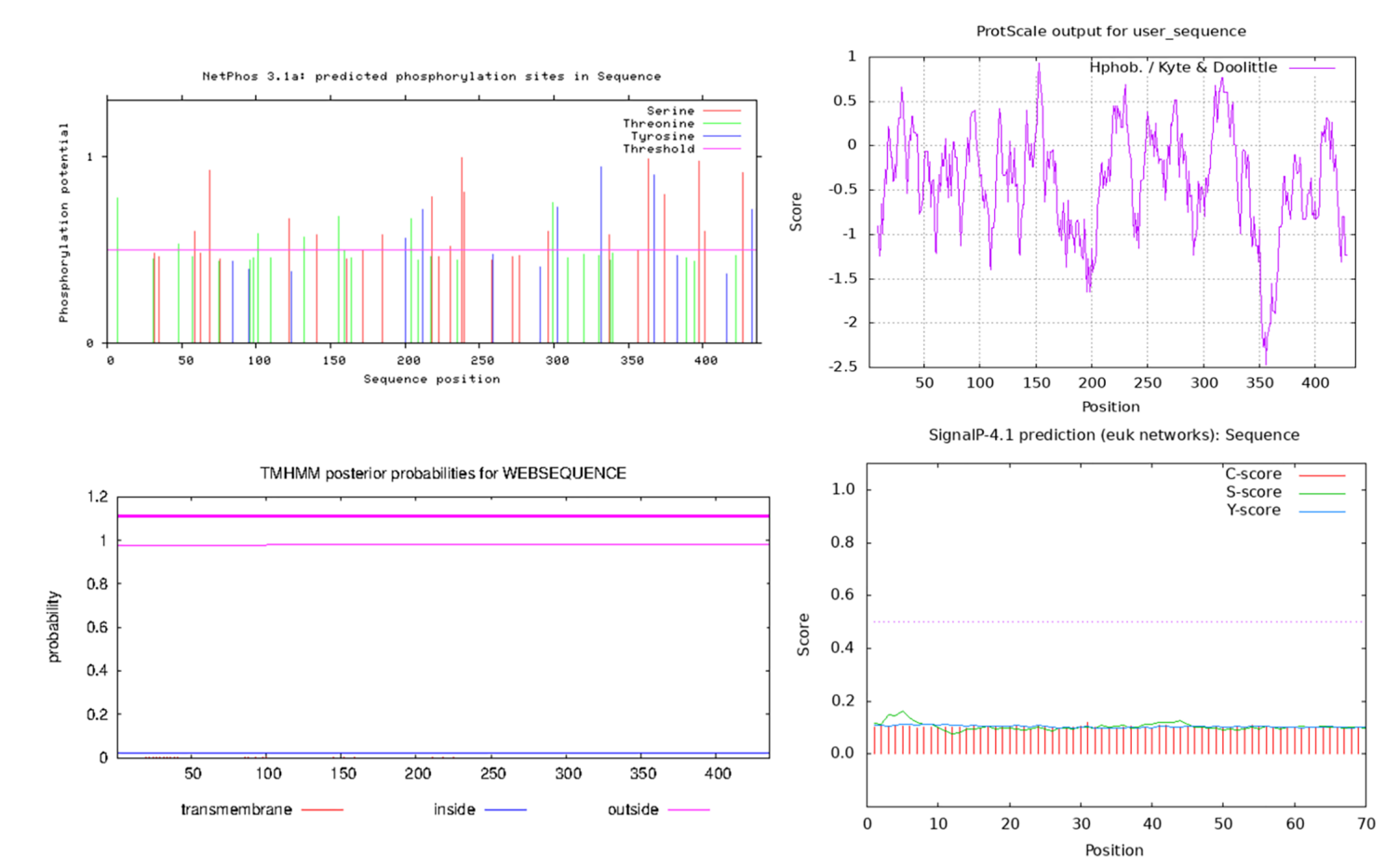
Figure S3.** Physicochemical properties of the *TaeRF1* protein. (A) Prediction analysis of the *TaeRF1* phosphorylation sites. (B) Prediction of the hydrophilicity or hydrophobicity of the *TaeRF1* protein. (C) Prediction of the transmembrane domain of the *TaeRF1* protein. The abscissa represents the position of each amino acid, and the ordinate represents the possibility of the residue being a transmembrane amino acid. The blue and red lines represent the intramembrane and extramembrane regions, respectively. (D) Signaling peptide prediction of the *TaeRF1* protein.

**C**

**A**

**D**

**B**


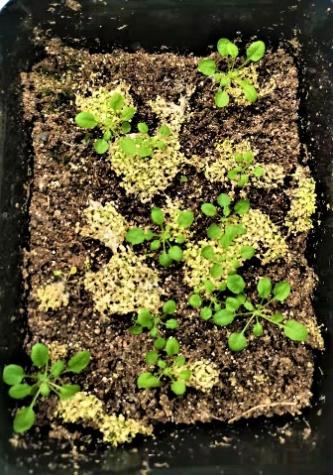

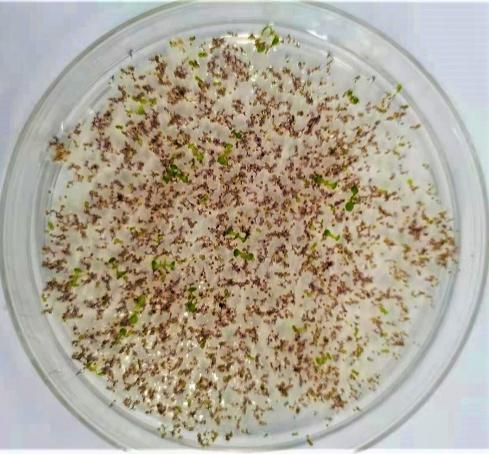


**C**

**A**

**D**

**B**

**E**


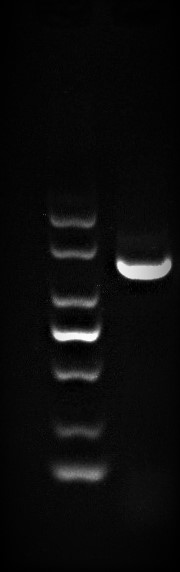


2000bp

750bp

M 1


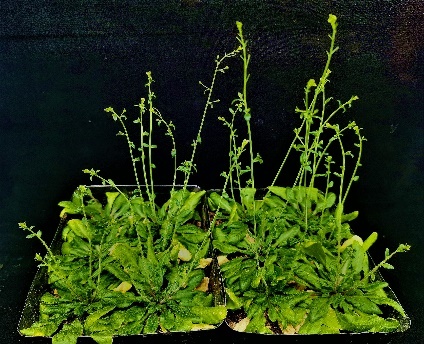

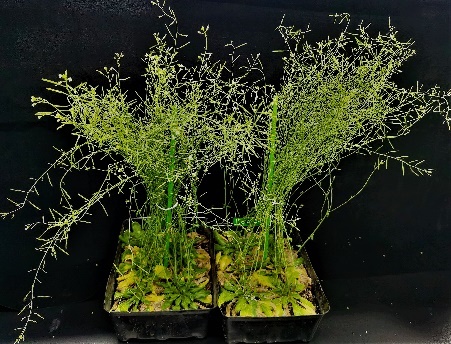

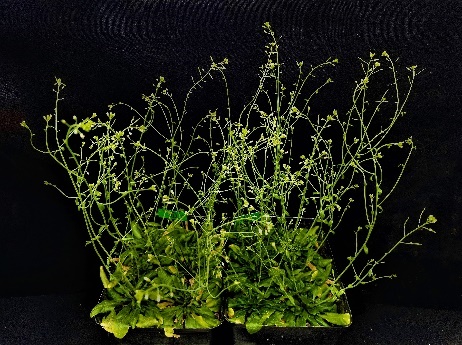


**Figure S4.** Genetic transformation and electrophoretic detection of positive-expression Arabidopsis plants. (A) Electrophoretic image of Agrobacterium. m: marker,1: TaeRF1. Genetic transformation (B) and resistance screening (C) of Arabidopsis Thaliana. (D) Electrophoretic detection of Arabidopsis positive plants. (E) Positive plant culture of Arabidopsis thaliana.


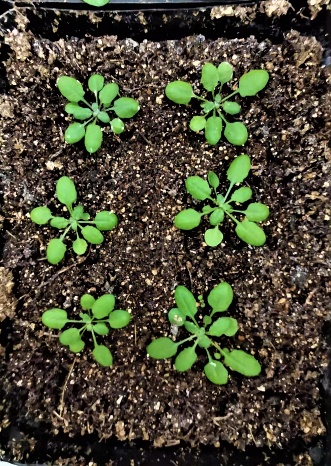

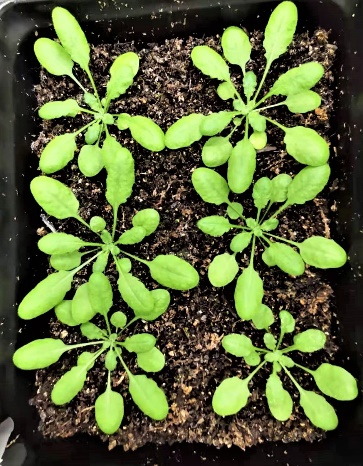

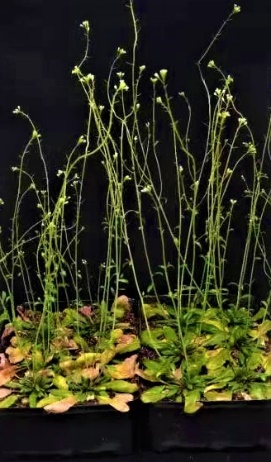

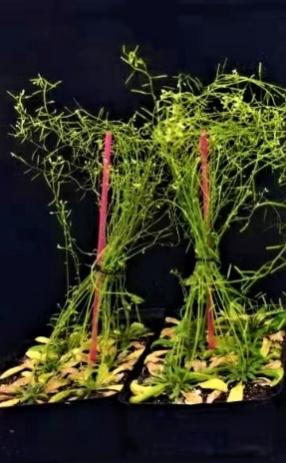


+ + M 1 2 3 4 5 6 7 8 9 10 11


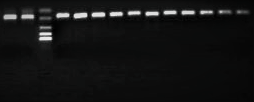

Supplement: Supplementary file 1 [file DataSheet_1.zip › Supplementary Material Presentation/Supplementary_Data_Figure S1-S4.docx]
