# Supplementary material for: Cloning of TaeRF1 gene from Caucasian clover and its functional analysis responding to low-temperature stress
Source: Front Plant Sci. 2022 Dec 20;13:968965. doi: 10.3389/fpls.2022.968965 (PMC9809470; doi:10.3389/fpls.2022.968965)
Supplement: Supplementary file 1 [file DataSheet_1.zip › Supplementary Material Presentation/Supplementary_Data_Table S1-S2.docx]

| **Primer name** | **Primer sequence(5’-3’)** |
| --- | --- |
| TaeRF1-F | ATGGCTGATGCTCATGATACTGA |
| TaeRF1-R | CTATTCGTCTTCATACACGCCTC |
| RG-F | TAATGCACCGAATCCCTCGT |
| RG-R | GAATGCAGATGACCGGTTGG |
| TaeRF1-TE-F | CGGGGGACTCTTGACGAGCTCATGGCTGATGCTCATGATACTGA |
| TaeRF1-TE-R | CATGTCGACTCTAGAGGATCCTTCGTCTTCATACACGCCTC |
| TaeRF1-OE-F | CAGTGGTCTCACAACATGGCTGATGCTCATGATAC |
| TaeRF1-OE-R | CAGTGGTCTCATACACTATTCGTCTTCATACACGC |
| Validation-F | GGATCACTCGATTTGTCAC |
| Validation-R | CAAGACCGGCAACAGGATTCAATC |
| AtCBF1-F | GCATGTCTCAACTTCGCTGA |
| AtCBF1-R | ATCGTCTCCTCCATGTCCAG |
| AtCBF2-F | TGACGTGTCCTTATGGAGCTA |
| AtCBF2-R | CTGCACTCAAAAACATTTGCA |
| AtCOR15B-F | GCAAAGCAGAGTGGTGTTGG |
| AtCOR15B-R | GGCCTCCTTCGTCTTATCCG |
| AtICE1-F | TCTTGTCTGCTCGGTCAC |
| AtICE1-R | AATCCCAGTCTCATCCATA |
| AtRD29A-F | CTCCATCAAGAAGCCATGAATTT |
| AtRD29A-R | CAACCATTCCTCCTCCTCCTT |
| AtCOR47-F | CAGTGTCGGAGAGTGTGGTG |
| AtCOR47-R | ACAGCTGGTGAATCCTCTGC |

**Table S1.** Primers used in this study.

**Table S2.** The CDS sequence and encoded amino acid sequence of TaeRF1*.*

| Name | Sequence |
| --- | --- |
| CDS  sequence | 1-50 ATGGC TGATG CTCAT GATAC TGATA AGAAC ATTGA GGTGT GGAAA ATCAA  51-100 GAAAT TGATC AAGGC TCTTG AAGCT GCTAG AGGAA ATGGG ACAAG TATGA  101-150 TTTCC CTTAT CATGC CACCA CGTGA TCAGA TTGCT CGTGT TACCA AGATG  151-200 CTTGG CGATG AGTTT GGAAC TGCTT CAAAC ATCAA AAGTA GGGTG AATCG  201-250 ACAGT CTGTG CTTGG TGCAA TCACT TCTGC TCAGC AGAGG CTTAA GCTTT  251-300 ATAAC AAGGT TCCTC CTAAT GGCCT CGTTT TGTAT ACTGG CACAA TTGTG  301-350 ACTGA TGATG GGAAG GAGAA AAAGG TGACC ATTGA TTTTG AACCA TTTAG  351-400 ACCTA TCAAC GCATC TCTCT ATCTT TGTGA CAACA AGTTT CACAC TGAAG  401-450 CTCTA AACGA GCTAC TGGAG TCTGA TGACA AGTTT GGATT TATTG TCATG  451-500 GATGG GAATG GCACT CTGTT TGGAA CTTTG AGTGG TAATA CAAGA GAGGT  501-550 GCTTC ACAAA TTCAG TGTGG ATCTC CCGAA GAAAC ATGGA AGAGG AGGGC  551-600 AATCA GCTCT ACGTT TTGCC CGTCT TCGTA TGGAG AAGCG TCATA ACTAT  601-650 GTGAG GAAGA CTGCT GAGCT CGCAA CCCAG TTTTA TATCA ATCCT GCTAC  651-700 CAGCC AGCCT AAT GT TTCTG GATTA ATTCT TGCTG GTTCA GCTGA TTTTA  701-750 AAACT GAGCT TAGTC AGTCA GATAT GTTTG ATCCA CGTCT TCAGG CAAAG  751-800 ATACT TAATG TTGTT GATGT ATCTT ATGGA GGGGA AAATG GGTTT AATCA  801-850 GGCTA TTGAA TTATC TGCTG AAATT CTGTC CAATG TCAAG TTTAT TCAGG  851-900 AGAAA CGCTT GATTG GAAAA TACTT TGAGG AAATC AGTCA GGATA CGGGG  901-950 AAGTA TGTCT TTGGT GTTGA TGATA CTCTA CAAGC TCTGG ATGCA GGAGC  951-1000 TGTCG AGACA CTTAT TGTCT GGGAA AATCT GGATA TGACT AGGTA TGTTT  1001-1050 TGAAA AATAG TACTA CTGGT GAAGT TGTCA TTAAG CACTT CAACA AGGAG  1051-1100 CAGGA AGCCA ACCAG AGCAA CTTTA GAGAT CCTGA AAGCA ATGCT GATTA  1101-1150 CGAGG TTCAG GAAAA GCTGT CTCTG TTGGA GTGGT TTGCA AATGA ATACA  1151-1200 GAAAG TTTGG ATGCA CTCTC GAGTT TGTCA CTAAT AAATC ACAAG AAGGT  1201-1250 TCACA GTTTT GCAGA GGTTT TGGTG GGATA GGTGG GATCT TGCGT TACCA  1251-1300 GCTTG ATATG AGAAC ATTTG ATGAT TTTTC TGATG ATGGA GGCGT GTATG  1301-1311 AAGAC GAATA G |
| amino  acid  sequence | MAD AHD TDK NIE VWK IKK LIK ALE AAR GNG TSM ISL IMP PRD QIA RVT KML GDE FGT ASN IKS RVN RQS VLG AIT SAQ QRL KLY NKV PPN GLV LYT GTI VTD DGK EKK VTI DFE PFR PIN ASL YLC DNK FHT EAL NEL LES DDK FGF IVM DGN GTL FGT LSG NTR EVL HKF SVD LPK KHG RGG QSA LRF ARL RME KRH NYV RKT AEL ATQ FYI NPA TSQ PNV SGL ILA GSA DFK TEL SQS DMF DPR LQA KIL NVV DVS YGG ENG FNQ AIE LSA EIL SNV KFI QEK RLIG KYF EEI SQD TGK YVF GVD DTL QAL DAG AVE TLI VWE NLD MTR YVL KNS TTG EVV IKHF NKE QEA NQS NFR DPES NAD YEV QEK LSL LEW FAN EYR KFG CTL EFV TNK SQE GSQ FCR GFG GIG GIL RYQ LDM RTF DDF SDD GGV YEDE |
