# Supplementary figures and images for: Cloning of TaeRF1 gene from Caucasian clover and its functional analysis responding to low-temperature stress
Source: Front Plant Sci. 2022 Dec 20;13:968965. doi: 10.3389/fpls.2022.968965 (PMC9809470; doi:10.3389/fpls.2022.968965)

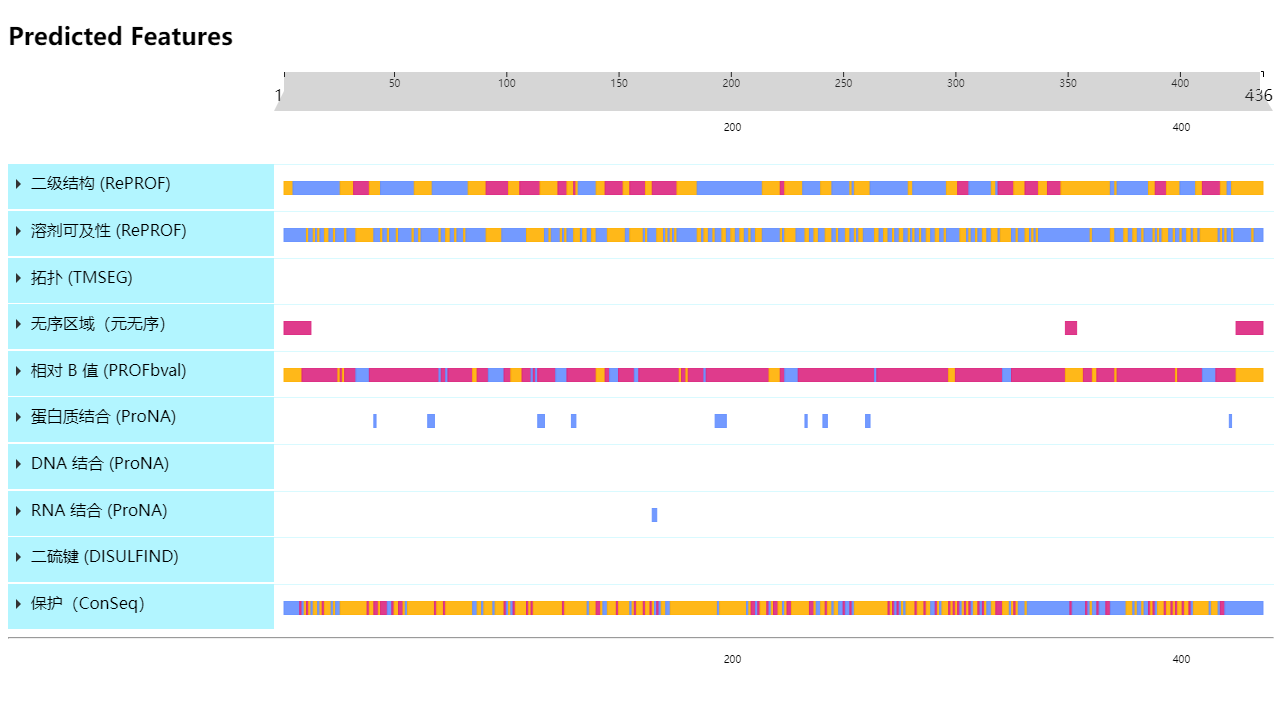

Supplement: Supplementary file 1 [file DataSheet_1.zip › Supplementary Material Presentation/Figure S2.png]

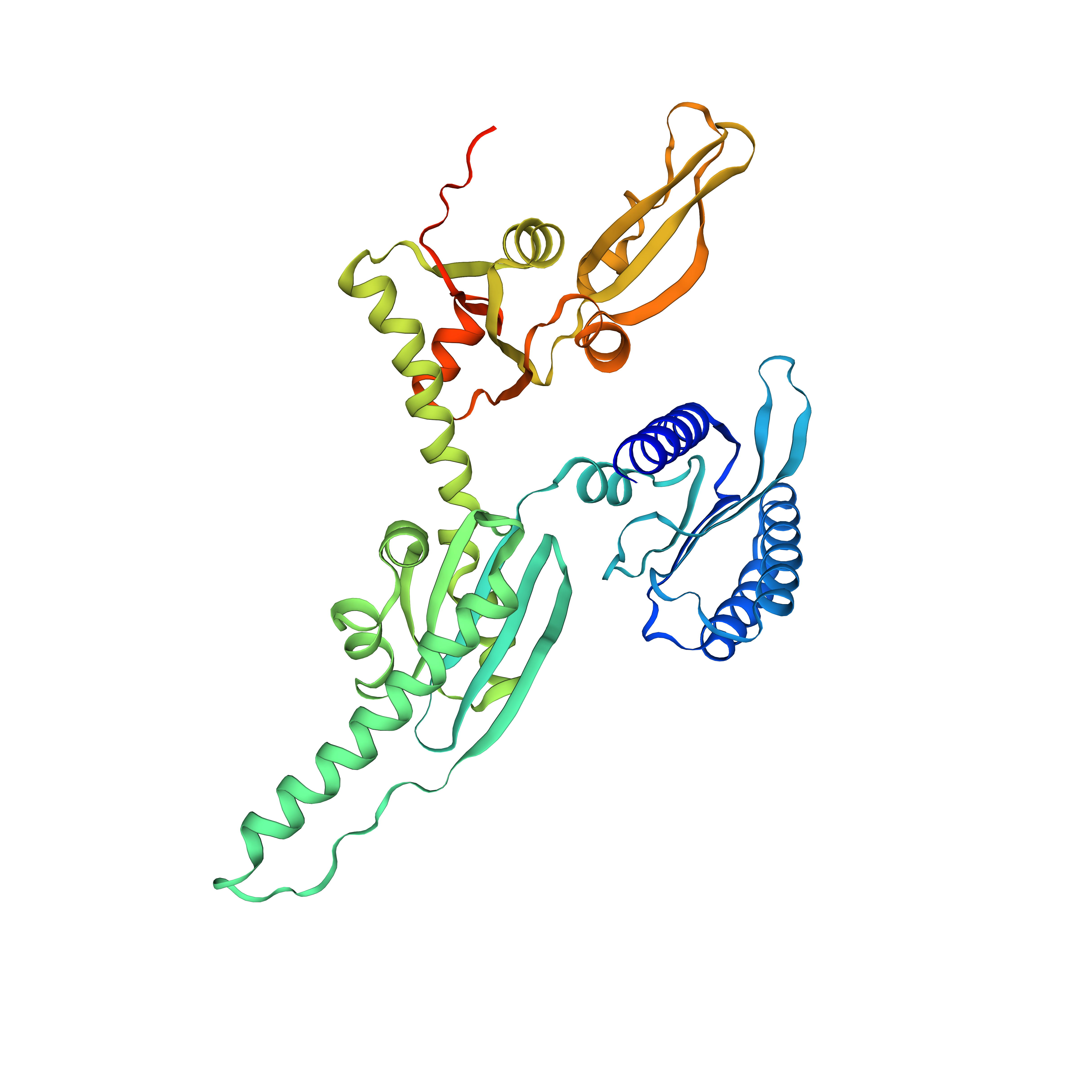

Supplement: Supplementary file 1 [file DataSheet_1.zip › Supplementary Material Presentation/Figure S2b.png]
